# Relationship between the rumen microbiome and liver transcriptome in beef cattle divergent for feed efficiency

**DOI:** 10.1186/s42523-024-00337-0

**Published:** 2024-09-20

**Authors:** Kate Keogh, David A. Kenny, Pamela A. Alexandre, Sinead M. Waters, Emily McGovern, Mark McGee, Antonio Reverter

**Affiliations:** 1 Animal and Bioscience Research Department, Teagasc, Animal & Grassland Research and Innovation Centre, Grange, Dunsany, Co. Meath, Ireland; 2 CSIRO Agriculture & Food, Queensland Bioscience Precinct, 306 Carmody Rd., St. Lucia, Brisbane, QLD 4067 Australia; 3 School of Biological and Chemical Sciences, Ryan Institute, University of Galway, Galway, Ireland; 4 Livestock Systems Research Department, Teagasc, Animal & Grassland Research and Innovation Centre, Grange, Dunsany, Co. Meath, Ireland

**Keywords:** Feed efficiency, Beef cattle, Gene co-expression network analysis

## Abstract

**Background:**

Feed costs account for a high proportion of the variable cost of beef production, ultimately impacting overall profitability. Thus, improving feed efficiency of beef cattle, by way of determining the underlying genomic control and selecting for feed efficient cattle provides a method through which feed input costs may be reduced whilst also contributing to the environmental sustainability of beef production. The rumen microbiome dictates the feed degradation capacity and consequent nutrient supply in ruminants, thus potentially impacted by feed efficiency phenotype. Equally, liver tissue has been shown to be responsive to feed efficiency phenotype as well as dietary intake. However, although both the rumen microbiome and liver transcriptome have been shown to be impacted by host feed efficiency phenotype, knowledge of the interaction between the rumen microbiome and other peripheral tissues within the body, including the liver is lacking. Thus, the objective of this study was to compare two contrasting breed types (Charolais and Holstein-Friesian) divergent for residual feed intake (RFI) over contrasting dietary phases (zero-grazed grass and high-concentrate), based on gene co-expression network analysis of liver transcriptome data and microbe co-abundance network of rumen microbiome data. Traits including RFI, dry matter intake (DMI) and growth rate (ADG), as well as rumen concentrations of volatile fatty acids were also included within the network analysis.

**Results:**

Overall, DMI had the greatest number of connections followed by RFI, with ADG displaying the fewest number of significant connections. Hepatic genes related to lipid metabolism were correlated to both RFI and DMI phenotypes, whilst genes related to immune response were correlated to DMI. Despite the known relationship between RFI and DMI, the same microbes were not directly connected to these phenotypes, the *Succiniclasticum* genus was however, negatively connected to both RFI and ADG. Additionally, a stepwise regression analysis revealed significant roles for both *Succiniclasticum* genus and *Roseburia.faecis sp.* in predicting RFI, DMI and ADG.

**Conclusions:**

Results from this study highlight the interactive relationships between rumen microbiome and hepatic transcriptome data of cattle divergent for RFI, whilst also increasing our understanding of the underlying biology of both DMI and ADG in beef cattle.

**Supplementary Information:**

The online version contains supplementary material available at 10.1186/s42523-024-00337-0.

## Background

Within beef production systems, feed costs alone account for up to 75% of the total variable cost of production [1], thus impacting overall profitability. Therefore, selecting and breeding cattle that are more feed efficient, for example through residual feed intake (RFI) phenotype, may increase production profitability by reducing feed input costs, whilst also contributing to the environmental sustainability of beef production. Moreover, feed intake (measured as average daily dry matter intake, DMI) and growth rate (measured as average daily gain, ADG) are important traits within the beef industry due to their direct and indirect effects on overall productivity and consequently profitability and sustainability. Thus, it is of interest for beef producers to rear faster growing animals with reduced dietary intake to optimise productivity of production systems [2]. Indeed, the aforementioned traits (DMI, ADG and RFI) have been shown to be moderately heritable [3–6], thus offering an opportunity to reduce feed costs through breeding cattle that are more feed efficient through genomic selection breeding programs. However, despite the clear benefit of breeding cattle that are feed efficient, evaluations of the molecular control regulating feed efficiency in beef cattle are not conclusive, such that key genes or genomic regions contributing to the trait are yet to be identified [1]. The contrasting results from molecular and genomic based studies across the literature are undoubtedly due to the multifaceted nature of the feed efficiency trait as well as the various confounding experimental parameters employed across studies such as breed, dietary management system and stage of development during which feed efficiency was evaluated. If reliable genomic selection processes are to be implemented for feed efficiency in beef cattle it is essential that genomic regions contributing to the trait are reliable across these various confounding factors.

The availability of nutrients for both growth and maintenance purposes in ruminants is dependent on the functionality of the rumen microbiome. Ruminants rely on the complement of bacteria, archaea and protozoa, amongst others, in the rumen microbiota for the degradation of feed and the production of microbial protein, vitamins and volatile fatty acids (VFAs), the latter of which provide up to 70% of the host’s energy requirements [7]. Research from our own group as well as others has identified links between the rumen microbiome and feed efficiency phenotype [8–13]. Moreover, Shabat et al. [14] reported that the rumen microbiome could predict variation in an animal’s feed efficiency phenotype and concluded that reduced rumen microbial community diversity may support a more feed efficient animal. However, similar to the identification of genomic regions associated with feed efficiency in beef cattle, results related to the contribution of the rumen microbiome to the feed efficiency phenotype are inconsistent, again, most likely due to the aforementioned confounding experimental parameters employed. Thus, it is important that such factors are considered and examined together [13]. Determination of the relationship between the rumen microbiome and host feed efficiency phenotype has the potential to not only facilitate the selection of cattle with enhanced nutrient utilisation, but also to enable the manipulation of the rumen microbiome to enhance its energy harvesting capacity [11, 14, 15].

The VFAs produced within the rumen following microbial degradation of feed are responsible for a large proportion of the host’s energy requirements. Furthermore, the liver, a highly metabolically active organ, has been shown to be responsive to dietary intake [16] as well as being affected by feed efficiency phenotype [2]. Thus, the objective of this study was to evaluate the interactions between the rumen microbiome, VFA concentrations and hepatic gene expression profiles with three production and efficiency traits of interest, namely DMI, RFI and ADG, through a network based systems biology analysis. In order to account for the confounding effects of both breed and dietary source, data utilised in this study (rumen microbiome, liver transcriptome, VFA and phenotype data) were derived from two contrasting breed types (Charolais and Holstein-Friesian) divergent for RFI across contrasting dietary phases (high concentrate and zero-grazed grass).

## Results

### Animal performance

Descriptive results pertaining to growth, dietary intake and RFI values are outlined in full in Higgins et al. [17]. Groups selected as divergent for RFI were significantly different from one another (*P* < 0.05). Across all dietary phases for each breed, High-RFI steers consumed more feed on average than their Low-RFI counterparts (*P* < 0.001), whilst there was no difference (*P* > 0.05) in ADG across groups for each breed and each dietary phase.

### Microbial sequencing analysis

Complete results related to microbial sequencing are presented in full in McGovern et al. [13]. Briefly an average of 272,460 (*±* 69,596) reads were generated from the rumen fluid samples. Following merging of sequences and quality filtering, an average of 217,817 (*±* 55,519) reads were retained. The average number of counts per sample that were assigned to an open taxonomic unit (post filtering) was 175,304 ± 74,272. Microbial taxa identified within the rumen fluid samples and utilised within the current study are presented in Additional Table 1.

### Liver gene expression

For the RFI, breed and diet contrasts, 12,161, 12,114 and 12,581 genes, respectively, were identified as expressed of which 608, 605 and 629 were classified as differentially expressed and used for subsequent co-expression network analysis. Of these differentially expressed genes, only 2 were common across the three contrasts, namely *SPP1*, which encodes a cytokine and *ABHD2* which encodes an acylglycerol lipase protein. Genes identified as differentially expressed and included in the co-expression network analysis are presented in Additional Table 2.

### Co-expression network analysis

**Fig. 1:**
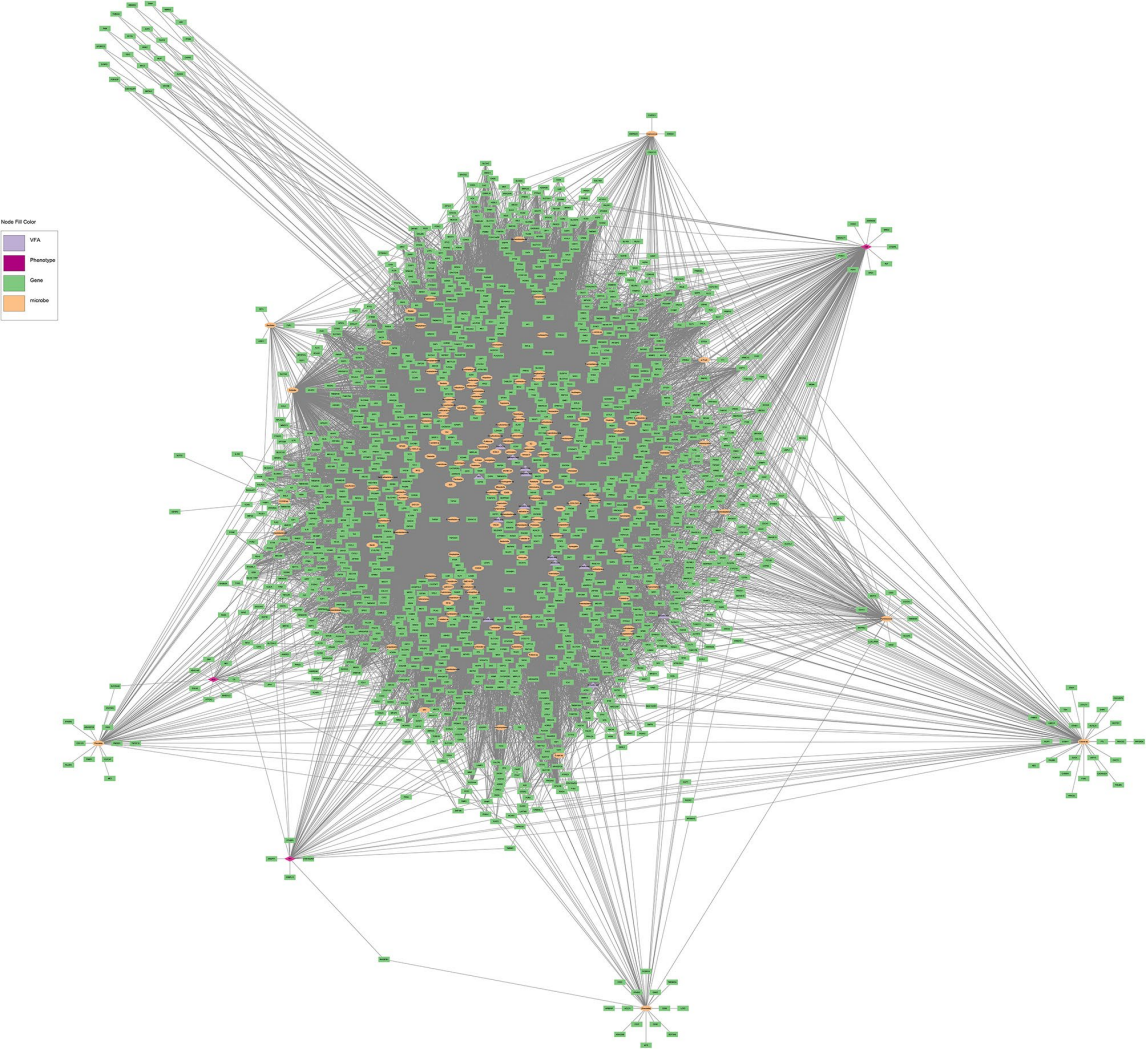
Gene co-expression network constructed using PCIT algorithm on 2,017 input nodes related to hepatic genes differentially expressed, rumen microbial taxa, volatile fatty acid concentrations and DMI, RFI and ADG phenotypes

**Fig. 2:**
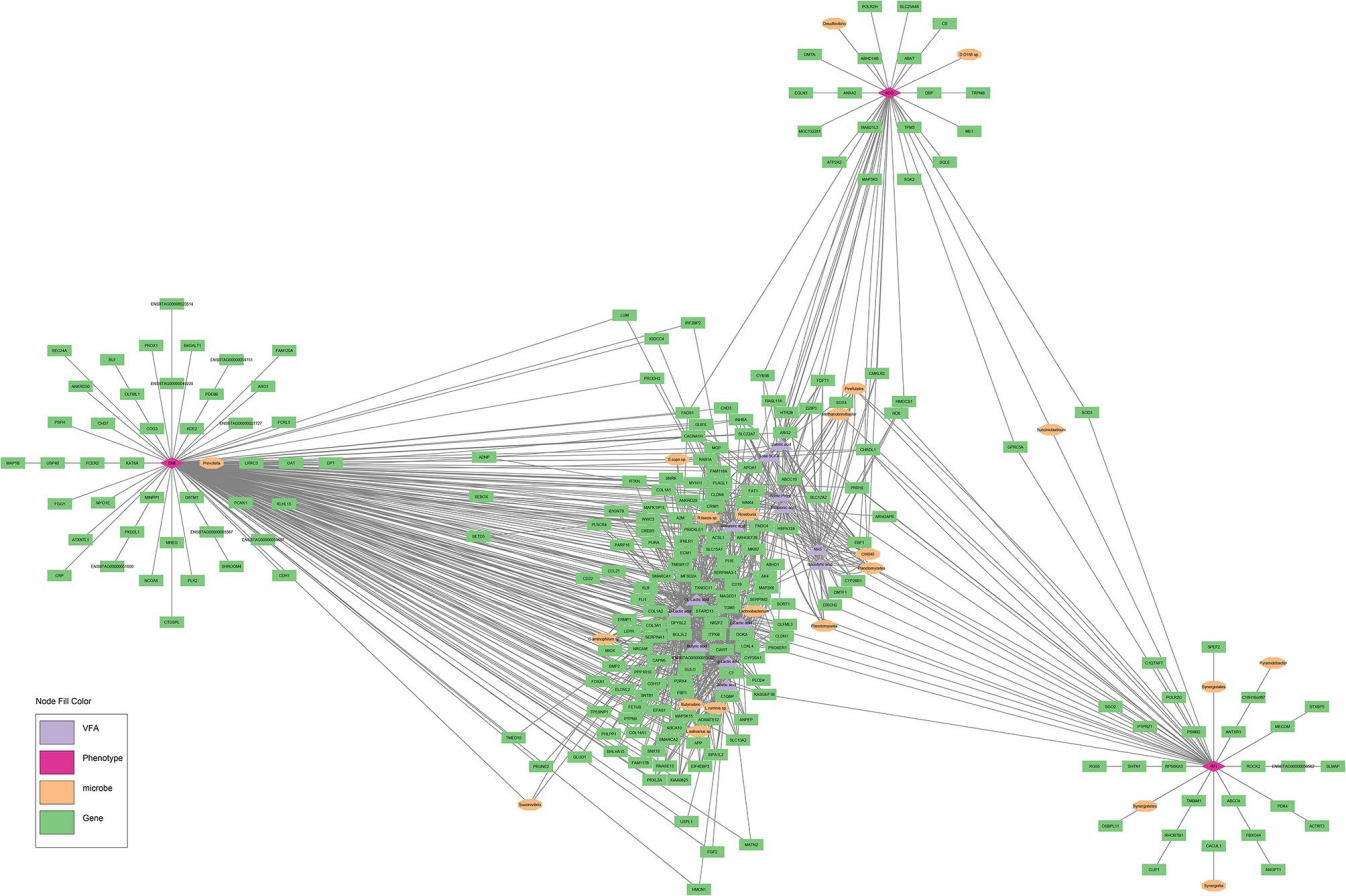
Genes, microbial taxa and volatile fatty acids directly connected to the DMI, RFI and ADG phenotypes

**Fig. 3:**
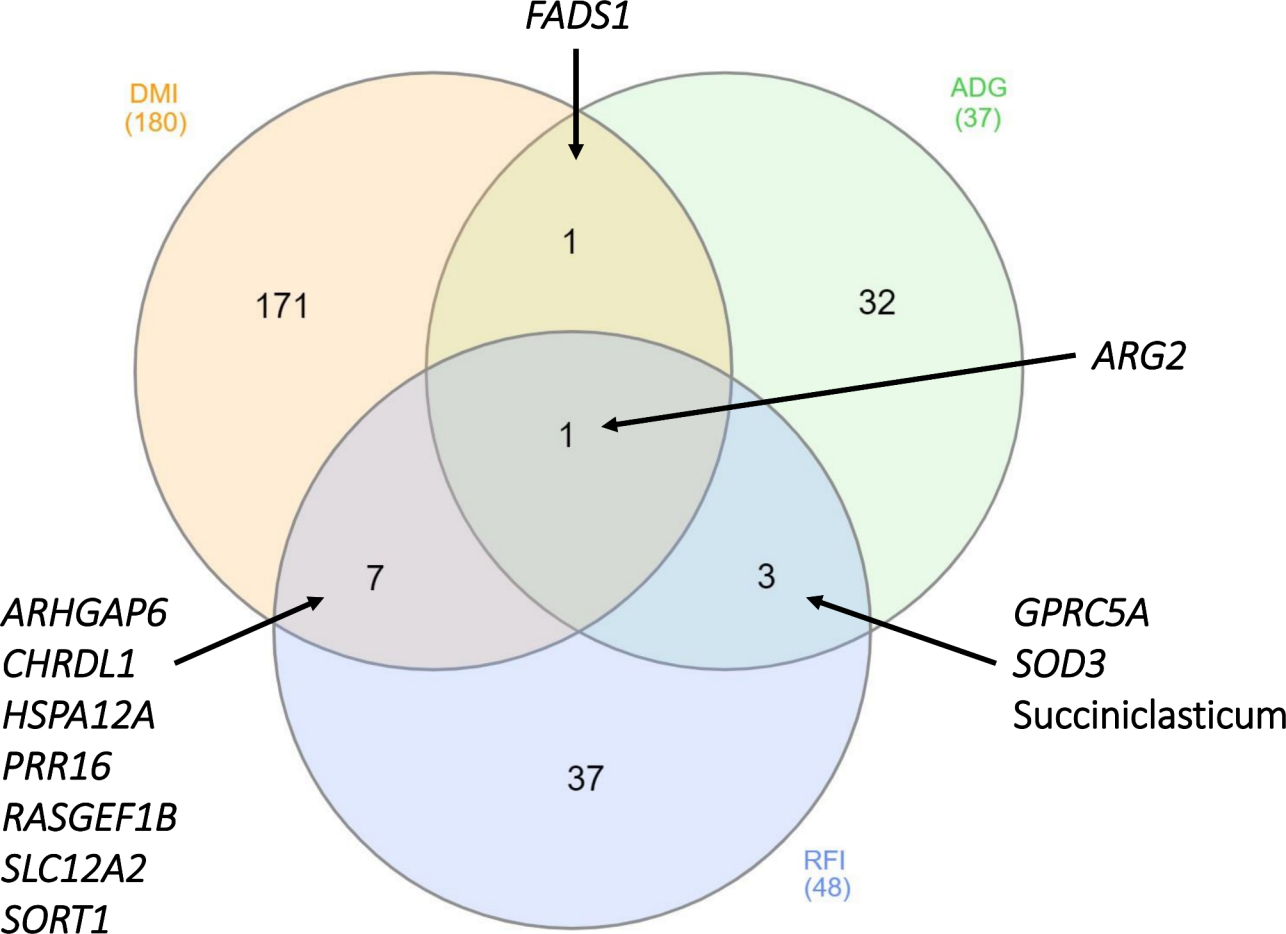
Venn diagram depicting commonality of first neighbour connections of the DMI, RFI and ADG phenotypes

**Fig. 4:**
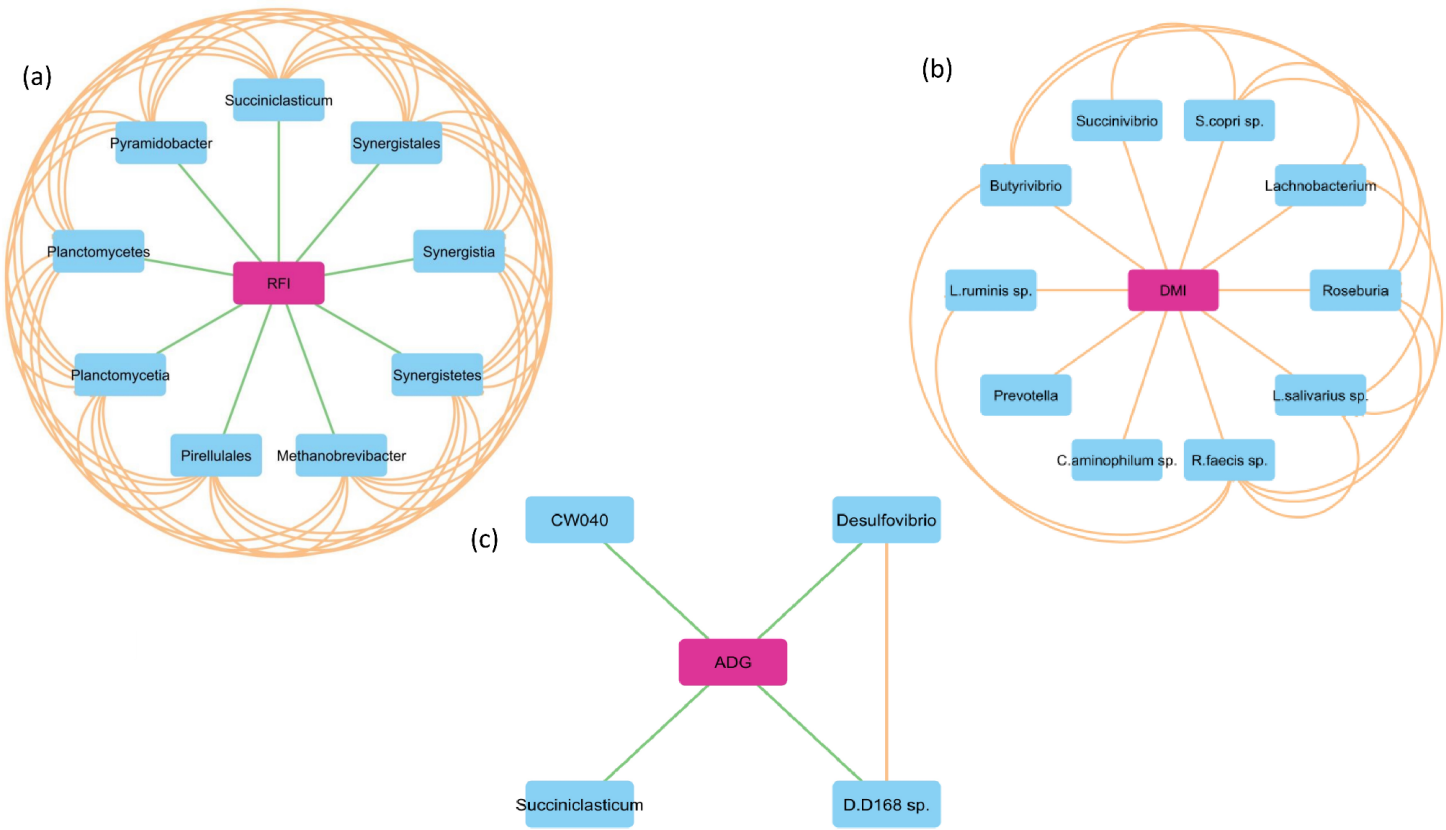
Microbial taxa directly interacting with (**a**) RFI, (**b**) DMI and (**c**) ADG. Orange lines depict positive connections, whist green lines indicate negative connections

Of the 2,017 nodes (1,842 differentially expressed genes, 159 microbial taxa, 13 VFA and 3 phenotypes) used for network analysis, 1,534  displayed significant correlations, resulting in a total of 35,549 significant (*P* < 0.05) connections between nodes (Fig. 1). Of the three phenotypes examined, DMI had the highest number of first neighbour connections, followed by RFI, with ADG displaying the fewest number of first neighbour connections (Table 1). Connections between hepatic genes accounted for the highest proportion of first neighbour connections for each of the three phenotypes, followed by microbial interactions, and VFA connections (Table 2). The co-expression network pertaining specifically to direct connections of the DMI, RFI and ADG phenotypes is presented in Fig. 2 and a comparison of nodes directly connected to DMI, RFI and ADG is provided in Fig. 3 and Additional Table 3. Direct connections between the phenotypes and microbial taxa revealed a lack of commonality between DMI and RFI, as well as DMI and ADG, with only the *Succiniclasticum* genus negatively connected to both RFI and ADG (Fig. 4). Details related to interactions of microbial taxa directly connected to DMI, RFI and ADG are presented in Table 3, along with enriched (*P* < 0.05) gene ontology and pathway analysis results. Full details related to interactions of microbial taxa connected to DMI, RFI and ADG are presented in full in Additional Table 4.

**Table 1:** Proportion of first neighbour nodes and direct connections pertaining to the DMI, RFI and ADG phenotypes

Phenotype	Number of first neighbour nodes	Number of direct connections	Percentage of first neighbour nodes from main network	Percentage of direct connections from main network
DMI	180	811	11.7%	2.3%
RFI	49	131	3.2%	0.37%
ADG	37	66	2.4%	0.19%

**Table 2:** Proportion of first neighbour phenotype nodes that are microbes, genes, VFAs or phenotypes

Node type	ADG	DMI	RFI
Number of interactions	37	180	49
Microbe	4 (10.8%)	10 (5.55%)	9 (18.4%)
VFA	2 (5.4%)	5 (2.8%)	0 (0%)
Phenotype	0 (0%)	1 (0.55%)	1 (2%)
Gene	31 (83.8%)	164 (91.1%)	39 (79.6%)

**Table 3:** Connections of microbial taxa directly interacting with DMI, RFI and ADG phenotypes

Microbe	Connections	Microbe	VFA	Gene	Enriched term
**DMI**					
-C.a*minophilum sp.*	60	6	5	48	Steroid biosynthesis Proteasomal processes
-*Butyrivibrio*	69	24	4	40	Nucleic acid binding
-*S.copri sp.*	57	12	4	40	Cholesterol biosynthesis
-*R.faecis sp.*	186	83	9	93	Cholesterol homeostasis
-*Lachnobacterium*	53	6	2	44	Transcription
-*Prevotella*	73	6		66	Carbohydrate metabolism
-*Roseburia*	187	86	9	91	Steroid biosynthesis Metabolic pathways
-*L.ruminis sp.*	65	19	4	41	Retinoid binding
-*L.salivarius sp.*	160	74	4	81	Steroid biosynthesis Metabolic pathways
-*Succinivibrio*	42	20	1	20	Plasma membrane
**RFI**					
-*Methanobrevibacter*	426	78	2	346	Metabolic pathways MAPK signaling Lipid transporter activity Catabolic processes
-*Pirellulales*	331	72	5	253	Ras signaling pathway Interferon signaling Steroid catabolic process Inflammatory response
-*Planctomycetes*	323	72	5	245	Interferon signaling
-*Planctomycetia*	331	72	5	253	Ras signaling pathway Interferon signaling Steroid catabolic process Inflammatory response
-*Pyramidobacter*	89	21		67	B-cell receptor signaling Chemokine signaling T-cell receptor signaling Immunodeficiency
-*Succiniclasticum*	43	16		25	Plasma membrane
-*Synergistales*	185	33		151	Hippo signaling Immunodeficiency
-*Synergistetes*	185	33		151	Hippo signaling Immunodeficiency
-*Synergistia*	185	33		151	Hippo signaling Immunodeficiency
**ADG**					
-*CW040*	217	72	6	138	Steroid biosynthesis Metabolic pathways Citrate cycle
-*D.D168 sp.*	26	5		20	Ras signaling pathway
-*Desulfovibrio*	27	4		22	Endoplasmic reticulum
-*Succiniclasticum*	43	16		25	Plasma membrane

Taxonomic levels for all microbes listed are presented in Additional Table 1

### Stepwise regression and correlation analyses

When each phenotype and their associated microbial connections were analysed separately through the regression analysis, 83% of the variation was accounted for through the *Pyramidobacter* and *Succiniclasticum* genera for the RFI phenotype. Similarly, the *Succiniclasticum* genus, together with the *CW040* order explained 73% within the ADG trait, whilst the *Butyrivibrio* and *Prevotella* genera, together with S.*copri sp.* and *R.faecis sp.* explained 89.9% of the variation within the DMI trait. When all microbes directly connected to the phenotypes examined were analysed together, 90% of the variation for RFI, DMI and ADG was explained by the following microbial taxa: the *Planctomycetes* phylum; *CW040* order; the *Prevotella*, *Roseburia*, *Succiniclasticum*, *Methanobrevibacter*, *Pyramidobacter*, *Butyrivibrio* genera and; *S.copri sp.*, *R.faecis sp.*, *L.ruminis sp.*, *C.aminophilum sp.* and *D.D168 sp.* (Table 4). Of the significantly correlated microbes, only the *Succiniclasticum* genus and *R.faecis sp.* were commonly contributing to variation across the three traits examined.

**Table 4:** Stepwise regression analysis results of RFI, DMI and ADG phenotypes with significantly correlated microbial taxa. For each phenotype the microbes listed account for 90% of the variation

RFI	DMI	ADG
*Prevotella*	*Butyrivibrio*	*Succiniclasticum*
*Roseburia*	*Prevotella*	*Methanobrevibacter*
*Succiniclasticum*	*Succiniclasticum*	*C.aminophilum*
*Methanobrevibacter*	*Planctomycetes*	*R.faecis sp.*
*Pyramidobacter*	*Aminophilum*	*D.D168 sp.*
*S.copri sp.*	*S. copri sp.*	*CW040*
*R.faecis sp.*	*R.faecis sp.*	
*Ruminis*		

Taxonomic levels for all microbes listed are presented in Additional Table 1

**Fig. 5:**
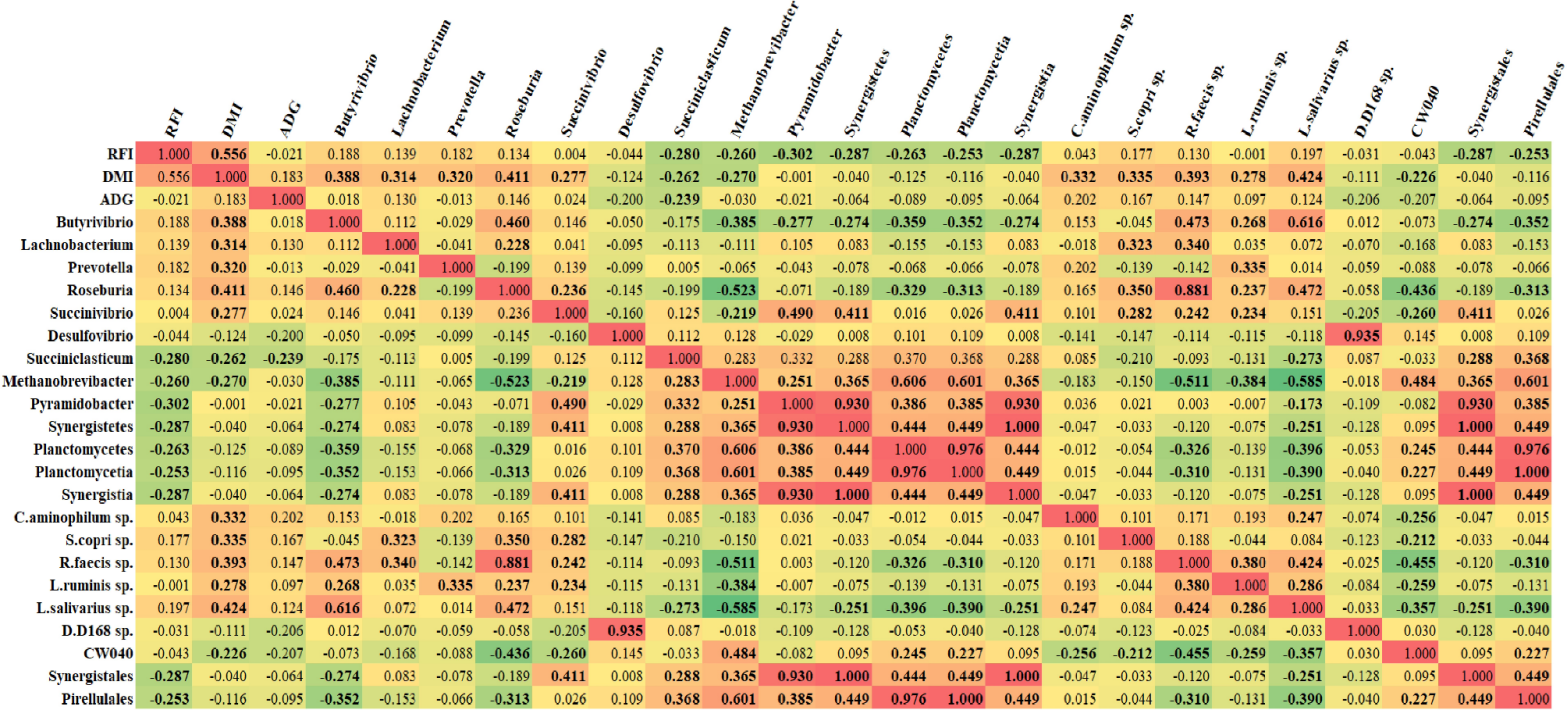
Results from correlation analysis between the RFI, DMI and ADG phenotypes and microbial taxa directly connected to the phenotypes examined. Green cells represent negative correlations, with positive correlations in red. Cells in bold font represent significant associations (*P* < 0.05)

Results of the correlation analysis undertaken in SAS are presented in Fig. 5. Correlation results followed the same pattern as per the co-expression network results for RFI (Fig. 4), with negative correlations (*P* < 0.05) apparent between RFI and the *Succiniclasticum* and *Pyramidobacter* genera, the *Synergistetes* and *Planctomycetes* phyla, *Planctomycetia and Synergistia* classes and *Synergistales* and *Pirellulales* orders. In addition to the positive connections evident through the co-expression network analysis, through the CORR procedure in SAS, negative associations (*P* < 0.05) were also apparent between DMI and the *Succiniclasticum* and *Methanobrevibacter* genera and the *CW040* order. Similar to both RFI and DMI, a negative association (*P* < 0.05) was also apparent between the *Succiniclasticum* genus and ADG, representing the only significant association between ADG and the microbial taxa from the correlation analysis.

**Fig. 6:**
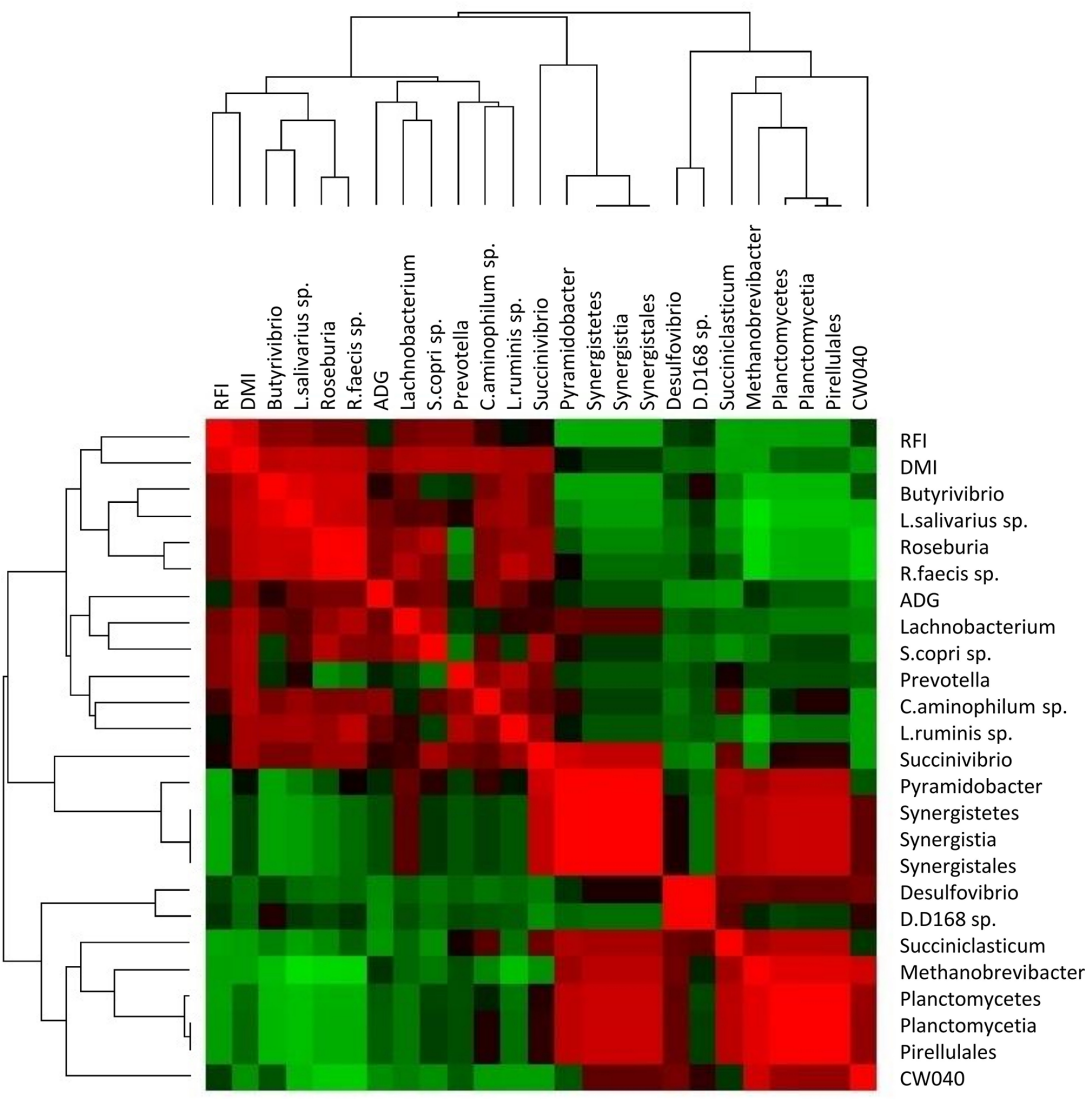
Heatmap of the hierarchical cluster analysis conducted in PermutMatrix between the ADG, RFI and DMI phenotypes with their first neighbour microbial connections derived from the PCIT results

Hierarchical clustering of the phenotypes and first neighbour microbial connections is presented in Fig. 6. From Fig. 6, it is evident that the RFI and DMI phenotypes were clustered with the *Roseburia* and *Butyrivibrio* genera, as well as with *L.Salivarius sp.* and *R.Faecis sp.* The ADG phenotype was separately clustered together with the *Lachnobacterium* and *Prevotella* genera, whilst also clustered with *S.Copri sp.*, *C.Aminophilium sp.* and *L.Ruminis sp.* Hierarchical clustering also revealed a relationship between members of the same clade, for example the *Pyramidobacter* genus, *Synergistetes* phylum, *Synergistia* class and *Synergistales* order were all clustered together, with the *Planctomycetia* class, *Planctomycetes* phylum and *Pirellulales* order also clustered together separately.

## Discussion

Within the published literature, biological processes related to immune function and lipid metabolism have been attributed to the underlying biology governing the RFI phenotype [17–21]. Indeed, results from the current study further corroborate that finding, where lipid metabolism related genes pertaining to the RFI differential expression contrast were connected to the RFI phenotype, whilst also connected to both DMI and ADG too. For example, RFI was positively connected to *CYP26B1*, whilst also negatively related to both *PDK4* and *OSBPL11*. Indeed, all three of these differentially expressed genes were pertaining to the RFI contrast, whilst *CYP26B1* was related to both RFI and diet contrasts, indicating a role for this gene in mediating the intersection of RFI with diet. *CYP26B1* encodes a member of the cytochrome P450 superfamily which are responsible for catalysing reactions involved in the synthesis of cholesterol, steroids and other lipids. Additionally, *CYP26A1*, which functions similarly to *CYP26B1* was also positively connected to the DMI phenotype. *OSBPL11* encodes an intracellular lipid receptor, which plays a role in regulating *ADIPOQ* and *FABP4* levels in differentiating adipocytes and is also involved in regulating adipocyte triglyceride storage. Whilst *PDK4* encodes a mitochondrial protein which functions in the regulation of both glucose and fatty acid metabolism. The relevance of the *PDK4* gene towards the RFI phenotype is further apparent through previous reports of this gene in relation to RFI, specifically *PDK4* was down-regulated in skeletal muscle tissue of efficient Holstein-Friesian bulls following a high-concentrate finishing diet [21], as well as in the liver tissue of efficient Charolais steers [17]. Of the lipid related genes connected to the DMI phenotype, all were pertaining specifically to the RFI differential expression contrast, whilst also being directly connected to the DMI phenotype, suggesting a role for these genes towards both RFI and DMI phenotypes. Moreover, a number of these genes have previously been implicated with variation in RFI, including *ASCL1* [20, 22]; *APOA1* [19, 23], *ELOVL2* [19] and *FADS1* [20]. The *FADS1* gene, which encodes a desaturase enzyme and regulates the unsaturation of fatty acids was also connected to ADG. Interestingly of the lipid metabolism genes connected to ADG (*HMGCS1*, *FDFT1*, *SQLE* and *FADS1*), all were pertaining to the diet differential expression contrast, with the exception of *FADS1*, highlighting the influence of contrasting diets with growth rates in beef cattle.

A role for altered immune response towards the RFI phenotype across various tissues throughout the body including the liver, has already been established [17–21]. Interestingly within the current study we did not observe such a relationship between the RFI phenotype and immune response genes, with the exception of, *C1QTNF7* which was negatively connected to RFI. However, although not strongly apparent within the RFI phenotype, a more pronounced relationship of immune response genes was evident with the DMI phenotype. This was apparent through connections between DMI and the following genes: *A2M*, *C1QBP*, *C7*, *CCL21*, *CD19*, *CD22*, *CRP*, *FCER2*, *FCRL5*, *FNDC4*, *HMCN1*, *IFNLR1* and *IRF2BP2*. Indeed, *A2M*, *CCL21* and *CRP* were previously reported as differentially expressed between cattle divergent for RFI [22, 23], suggesting the importance of these genes to both the RFI and DMI traits. The relationship between the DMI phenotype and immune response is further established through the connection between the *Prevotella* genus and *S.copri sp. (Segatella copri sp.* formerly *Prevotella copri sp.)* of bacteria within the rumen microbiome, both of which have been implicated in immune function. For example, high abundance of *P.copri sp.* was correlated with increased concentrations of serum metabolites related to chronic inflammation in the gut of pigs in the data of Chen et al. [24]. Whilst the *Prevotella* genus was reported to be associated with gut mucosal inflammation in mice [25]. Moreover, a positive relationship was apparent in the current study between the *Prevotella* genus and DMI, whilst Jiang et al. [26] and Carberry et al. [8] both conversely reported negative correlations between relative abundance of ruminal *Prevotella* genus. Moreover, both *Prevotella* genus and S.*copri sp.* have been related to RFI within various species too. For example, Jiang et al. [27] reported that *P.copri sp.* was one of the most abundant microbes in low-feed efficiency pigs and also noted the importance of that particular species to feed efficiency during daily-phase feeding strategies in pigs [28]. Furthermore, in cattle, Brooke et al. [29] suggested that *P.copri sp.* may be a potential microbial marker for the identification of cattle with improved feed efficiency in their life-span and in the production cycle. Additionally, Carberry et al. [8] reported an effect of RFI phenotype on ruminal abundance of *Prevotella* genus, irrespective of the varied diets offered in that study, but did note greater abundance of *Prevotella* genus when cattle were offered a low forage diet compared to a high forage diet. Conversely though, Lopes et al. [30] reported that the correlation between the *Prevotella* genus and feed efficiency was dependent on the diet offered as well as the specific *Prevotella spp.* Indeed, different *Prevotella spp.* have been associated with both higher and lower feed efficiency in cattle and sheep [29, 31–35]. Furthermore, Zhou et al. [36] reported that the *Prevotella* genus was the most abundant genus in both rumen content-associated and epithelial tissue-attached bacterial communities suggesting a role for this genus in serving as a marker for host RFI classification. Additionally, Jewell et al. [9] reported that the abundance of specific Prevotella *spp.* is host specific, with Yang et al. [37] suggested that *Prevotella* may be a key microbe increasing host feed intake, suggesting that *Prevotella spp.* could promote the host’s appetite and decrease feed efficiency. Overall, results indicate that the role of varied immune response in relation to RFI may also be attributed to variation in DMI. 

Despite the known relationship between RFI and DMI, namely RFI being computed after accounting for DMI, results from the current study highlight differential connections between these two phenotypes and ruminal microbiome taxa, whereby microbial taxa directly connected to RFI were different to those directly connected to DMI. For the DMI phenotype all direct connections between DMI and microbial taxa were positive, with interactions amongst these specific taxa also positive. Conversely though, for the RFI trait, all connections between RFI and microbial taxa were negative, whilst connections between the RFI taxa were positive. Overall suggesting differential microbial responses, despite the relationship between the two traits.

The *Butyrivibrio* genus, which was positively related to DMI in the current study, is involved in a number of ruminal functions in addition to butyrate production including fibre degradation, protein breakdown, biohydrogenation of lipids and the production of microbial inhibitors. Of particular importance to ruminant digestion, and therefore productivity is the contribution of this bacteria to the degradation of plant structural carbohydrates, principally hemicellulose. Together with the *Prevotella* genus, both *Butyrivibrio* and *Prevotella* genera are among the most abundant bacteria found within the rumen and have important functions in the metabolism of proteins and peptides [34]. Both of these genera breakdown protein and carbohydrates in feed, undertake *de novo* peptide synthesis and use products of cellulose degradation from other cellulotyic bacteria as an energy source. In addition to being related to DMI, hierarchical clustering analysis revealed a relationship between the *Butyrivibrio* genus with RFI. Indeed, within the context of feed efficiency, Myer et al. [10] and McGovern et al. [12] reported greater abundance of the *Butyrivibrio* genus in efficient steers and a negative correlation with RFI, respectively. Conversely, Jewell et al. [9] reported greater abundance in the rumen of High-RFI dairy cows, indicating a positive relationship between RFI and rumen *Butyrivibrio* genus. The relative importance of the *Butyrivibrio* genus towards feed efficiency is also apparent in monogastrics, specifically the *Butyrivibrio* genus was strongly correlated with feed efficiency in pigs during the weaning phase, which the authors attributed to potentially be due to an enhanced ability to ferment complex carbohydrates [38]. Similarly, Kubasova et al. [39] also reported greater abundance of *Butyrivibrio* genus in fecal samples of pigs. Although the *Butyrivibrio* genus is a primarily butyrate producing bacteria, results from the current interaction study reported direct connections with only the lactic acid VFAs, which in turn were connected to hepatic genes involved in functions related to gene expression, lipid metabolism, growth, as well as protease activity, which given the function of the *Butyrivibrio* genus towards protein breakdown is of interest. Specifically, genes involved in the regulation of peptidase activity (*PI16*) as well as those with an associated protease function (*CAPN6*, *SERPINA1*, *SERPINA3-1*) were connected to lactic acid VFAs within the rumen. Moreover, of these, *PI16*, *SERPINA1* and *SERPINA3* were previously reported as differentially expressed in the RFI based datasets of Keogh et al. [21], Weber et al. [22], and Alexandre et al. [23]. The lactic acid VFAs were also connected to the leptin receptor gene (*LEPR*) highlighting the role of leptin towards mediating satiety status within the body. Moreover, the *LEPR* gene was differentially expressed through the breed contrast, highlighting differential hepatic expression of this gene between Charolais and Holstein-Friesian steers. Additionally, genes involved in TGF-beta growth signaling (*BMP2*, *CRM1A*, *FNDC4* and *HMCN1*) were also connected to ruminal lactic acid concentrations, which was of interest as Alexandre et al. [23] previously reported *TGFB1* as a key regulator for feed efficiency in skeletal muscle of Nellore cattle.

Similar to the *Butyrivibrio* genus, the *R.faecis sp.* is also a primarily butyrate producing bacteria. Indeed, although correlated with DMI in the current study, as well as clustered with both DMI and RFI through the hierarchical clustering analysis, *R.faecis sp.* was previously associated with growth rate in pigs, with a greater abundance of this microbe in pigs supplemented with a carbohydrate complex diet, which the authors of that study suggested could have accounted for the improved feed efficiency observed in those animals [40]. However, through the regression analysis in the current study, *R.faecis sp.* was identified, together with the *Succiniclasticum* genus, as playing a role in determining variation in the RFI, DMI and ADG traits examined in this study. Volatile fatty acids connected to *R.faecis sp.* (lactic acid, ammonia, valeric acid, acetic:propionic, isobutyric acid and total-SCFA), were also connected to hepatic genes involved in processes related to gene expression, growth, immune and lipid metabolism. In the current study, the *Roseburia* genus was positively associated with DMI, this microbe utilises carbohydrates for growth and its abundance is known to increase with greater proportions of concentrates within the diet [32, 41]. Similarly, Li et al. [42] and Ellison et al. [33] reported greater abundance of the *Roseburia* genus in cattle fed a high-energy diet and lambs fed a concentrate diet, respectively, indicating a role for this bacterium depending on diet composition. Additionally through its connections with isobutyric acid, the *R.faecis sp.* was connected to *CMKLR2* and *ND6* which encode proteins involved in adipokinetic hormone activity and glucose homeostasis as well as a mitochondrial gene, respectively, potentially suggesting a role for *R.faecis sp.* in mediating hepatic glucose homeostasis and mitochondrial function as a consequence of dietary intake. Moreover, *CMKLR2* was specifically pertaining to the diet contrast, whilst *ND6* was differentially expressed in both diet and RFI contrasts, overall highlighting the effect of diet on these genes related to *R.faecis sp.* abundance. Whilst a role for these genes related to RFI is already established through their differential expression within the published literature [23, 43].

The *Synergistetes* phylum was negatively connected with RFI in the current study. Similarly, McLoughlin et al. [44] also reported a negative correlation between this phylum and feed efficiency in the solid rumen fraction of sheep. Additionally, McCormack et al. [45] also reported a role for this microbe towards variation in RFI in pigs. In addition to the negative relationship between the *Synergistetes* phylum and RFI; class, order and genus members of this clade were also negatively connected to RFI. Moreover, the members of this specific phylum were all positively connected to each other. Specifically other members of this clade negatively connected to RFI included the *Synergistia* class, *Synergistales* order and *Pyramidobacter* genus. Indeed, the relationship between the members of this specific phylum was apparent through the PCIT; correlation and; hierarchical clustering analyses undertaken. Of these microbial taxa, the *Pyramidobacter* genus has previously been implicated towards variation in feed efficiency. For example, McLoughlin et al. [44] identified positive associations between the relative abundance of the *Pyramidobacter* genus and feed conversion ratio in sheep, whilst also reporting a negative association with ADG. Similarly in Simmental bulls, McGovern et al. [12] identified an association between RFI and abundance of the *Pyramidobacter* genus. Whilst in pigs McCormack et al. [45] and Kubasova et al. [39] identified an association between the *Pyramidobacter* genus and RFI in the cecal digesta of pigs and reported greater abundance of the *Pyramidobacter* genus in the fecal microbiota of Low-RFI pigs compared to High-RFI pigs, respectively. Similar to the *Synergistetes* phylum, the *Planctomycetes* phylum as well as class sub-member (*Planctomycetia*), and order member (*Pirellulales*) were also negatively correlated with RFI, whilst positively correlated with one another. Indeed, the *Planctomycetes* phylum was shown to be altered in both pigs and hens divergent in feed efficiency potential [45, 46]. In cattle, Freetly et al. [47] reported an effect of ADG on abundance of the *Planctomyetes* phylum, *Planctomycetia* class and *Pirellulales* order in the rumen of beef cattle, with results from this current study highlighting a role for these microbes towards RFI in beef cattle.

Of all the microbes directly connected to the phenotypes examined in this study only one was commonly significantly correlated across more than one phenotype. Specifically, the *Succiniclasticum* genus was negatively correlated with both RFI and ADG. Moreover, through the regression analysis, the *Succiniclasticum* genus was observed to contribute to variation in all three traits examined in this study. A role for the *Succiniclasticum* genus towards variation in feed efficiency has been established previously within the published literature, for example, in a study by Myer et al. [10] more efficient steers were observed to have greater abundance of succinate producing bacteria including the *Succiniclasticum* genus. Auffret et al. [48] also reported significantly greater abundance of the *Succiniclasticum* genus in high feed efficiency beef cattle. Conversely though, Manzanares-Miranda et al. [49] reported lower abundance of the *Succiniclasticum* genus in Low-RFI bulls. Interestingly, an effect of diet on the abundance of *Succiniclasticum* genus is evident within the literature. For example, McCann [50] reported greater proportions of the *Succiniclasticum* genus in steers consuming low quality forage, however the same authors observed abundance to be mostly undetected in forage diets but more abundant in a high grain diet [32]. Additionally, McCabe et al. [51] reported alterations to the abundance of the *Succiniclasticum* genus dependent on the amount of feed consumed, whilst Luo et al. [52] showed that a high concentrate diet increased the abundance of the *Succiniclasticum* genus. Moreover, there is also evidence for an effect of breed on ruminal abundance of *Succiniclasticum* genus. For example, the *Succiniclasticum* genus was higher in the High-RFI beef cattle in Li et al. [42], however this difference was only apparent in Charolais and not in the other breeds examined in that study. Similarly, in sheep, McLoughlin et al. [53] identified higher abundance of the *Succiniclasticum* genus in the Connemara breed of sheep compared to other breeds. Together these results indicate towards a role for the *Succiniclasticum* genus towards the phenotypes examined in this study, however the contribution may be dependent on both individual genotype as well as the dietary management system in place.

## Conclusions

The rumen microbiome influences the availability of nutrients for subsequent growth purposes through the degradation of ingested feed. Equally the liver is a highly metabolically active organ and both the rumen microbiome and liver have been shown to be affected by RFI phenotype as well as dietary intake. Results from this study highlight the interaction amongst the rumen microbiome, VFA concentrations and hepatic gene expression profiles with three production and efficiency traits of interest, namely DMI, ADG and RFI. Results from this interactive study show a clear relationship between hepatic genes related to lipid metabolism towards RFI, whilst genes with an associated immune function were reported as primarily related to DMI. This study also indicated towards differential microbiome interactions between RFI and DMI despite the known correlation between these two traits, whilst the *Succiniclasticum* genus was identified as the only microbe connected to more than one trait, namely ADG and RFI. However, results highlight a potential role for both the *Succiniclasticum* genus and *R.faecis sp.* towards RFI, DMI and ADG phenotypes in beef cattle. Taken together, this study provides insights into the interaction amongst rumen microbiome and hepatic gene expression, which may be contributing to the underlying biology of DMI, ADG and RFI in beef cattle.

## Methods

### Animal management and phenotype collection

The animal model utilised in this study was conducted as part of a larger research programme designed to investigate the within-animal repeatability of feed intake, growth and feed efficiency in two contrasting breeds (Charolais and Holstein-Friesian) of beef steers, which were offered contrasting diets over separate dietary intake test periods [54, 55]. Details related to the original animal model are described previously in Higgins et al. [17] and are only briefly outlined here. Charolais (*n* = 90) and Holstein-Friesian (*n* = 77) steers were offered contrasting diets over different stages of development as follows: (i) a high-concentrate diet during the growing phase; (ii) zero-grazed grass diet during the growing phase and; (iii) high-concentrate diet during the finishing phase. Charolais steers were on average 373 (*±* 18) days of age and weighed 485 (*±* 38) kg, whilst Holstein-Friesian steers were 399 (*±* 7.6) days of age and weighed 401 (*±* 43.3) kg at the start of the trial. Upon completion of a dietary adaptation period, lasting 14 days, individual animal intakes were recorded (using an electronic Calan gate system; American Calan Inc., Northwood, NH, USA) over the three feeding phases, which each lasted for 70 days. Steers were weighed at the beginning and end of each dietary phase as well as on a fortnightly basis throughout. All steers were offered the same concentrate diet *ad libitum* during each of the two high-concentrate phases, with a restricted allowance of grass silage also provided. For the interim zero-grazed grass phase, steers were individually offered fresh herbage, harvested twice daily from *Lolium perenne* dominant swards, *ad libitum*. All steers had unrestricted access to fresh, clean drinking water. Upon completion of each dietary phase, individual RFI values were determined within breed for all steers as previously described in Higgins et al. [17], and animals were ranked as either High-RFI or Low-RFI, selecting the highest High-RFI and lowest Low-RFI for subsequent analyses.

### Rumen digesta sampling and sequencing

Full details related to rumen digesta sampling and subsequent sequencing analysis are described in full in McGovern et al. [13]. At the midpoint of each dietary phase, following a dietary adaptation period, a single rumen fluid sample was collected from all steers via stomach intubation (Flora Rumen Scoop, Profs-Products, Guelph, Canada). All samples were harvested approximately 2–4 h post-feeding, and were immediately snap frozen in liquid nitrogen and subsequently stored at -80 °C, pending further analysis. Ten rumen digesta samples per High- and Low-RFI groups from each breed and diet were used for subsequent microbial DNA isolation, with the exception of the Low-RFI Charolais and High-RFI Holstein-Friesian steers during the zero-grazed grass diet and the Low-RFI Holstein Friesian steers during the second high-concentrate diet, where only 9 samples were available for each. Frozen rumen liquid samples (20 g) was homogenised to a fine powder under liquid nitrogen using a pestle and mortar and stored at -80 °C. Approximately 250 mg of the homogenised frozen powder was then used for DNA isolation, which was undertaken using the repeated bead beating and column purification method [56]. The quality of the resultant DNA samples was assessed on an agarose gel, with DNA yield and purity also assessed on a Nanodrop 1000 spectrophotometer. Amplicon libraries were prepared through PCR amplification, targeting the V4 region of the 16 S rRNA gene in both bacteria and archaea. Full details of library preparation are previously outlined in McGovern et al. [13]. Amplicon generation was validated through visualisation on an agarose gel. Amplicons were pooled in equal concentrations and gel purified to remove unwanted products using the Qiagen Gel Extraction Kit (Qiagen, Manchester, UK). The pooled purified libraries were measured for purity and quantity on the Nanodrop 1000 spectrophotometer and further quantified using the KAPA SYBR FAST universal kit with Illumina Primer Premix (Roche Diagnostics, West Sussex, UK). The library pool was then diluted and denatured according to the Illumina MiSeq library preparation guide. The sequencing was conducted using 500 cycle MiSeq reagent kits (Illumins, San Diego, CA, USA). The sequencing reads generated were imported into Qiime2 [57], where the DADA2 pipeline [58] was used for the detection of operational taxonomic units [59]. Taxonomy was assigned using a naïve Bayes classifier trained on the RefSeq database [60]. Sequence read files associated with this analysis are available through the NCBI Sequence Read Archive (Accession no. PRJNA483745).

### Rumen fermentation profiling

The concentration of ruminal VFA composition was measured using a gas chromatograph (model 3800 Varian gas chromatograph) as per McGovern et al. [13]. The concentration of the following acids was determined: acetic, propionic, isobutyric, butyric, isovaleric, valeric, total short chain fatty acids, acetic:propionic acid ratio; D-lactic acid; L-lactic acid; DL-lactic acid; g-lactic acid and ammonia (NH_3_).

### Liver tissue sample collection and RNA-sequencing

Liver tissue sample collection and RNA-sequencing methodology is described in full in Higgins et al. [17]. Briefly, at the end of each dietary phase and within breed, steers were ranked as either High-RFI (feed-inefficient; *n* = 12) or Low-RFI (feed-efficient; *n* = 12) and were subsequently used for the collection of liver tissue biopsies. All steers selected for biopsy collection were administered a local anaesthetic (5 ml; Adrenacaine, Norbrook Laboratories, Ireland Ltd.) to the biopsy site location. Following anaesthetisation, liver tissue was harvested through percutaneous punch between the 11th and 12th ribs as previously described by McCarthy et al. [61]. Care was taken to ensure that all samples were consistently harvested from the same location from each animal. All instruments used for biopsy collection were sterilised, washed with 70% ethanol and treated with RNaseZap (Ambion, Applera Ireland, Dublin, Ireland), prior to use. Following collection, all tissue samples were washed with sterile DPBS and immediately snap frozen in liquid nitrogen before subsequent storage at -80 °C pending further processing.

Full details related to RNA isolation and subsequent RNA sequencing and bioinformatic analysis are provided in Higgins et al. [17] and are only summarised here. Briefly, 50 mg of liver tissue from each biopsy sample was used for the isolation of total RNA. RNA was isolated from tissue samples in 3 ml of QIAzol reagent using a rotor-stator tissue lyser (Qiagen, UK). RNA was subsequently precipitated and purified using the RNeasy plus Universal kit (Qiagen, UK) according to the manufacturer’s instructions. Quality and quantity of RNA isolated were determined using the RNA 6000 Nano Lab Chip kit (Aglient Technologies Ireland Ltd., Dublin, Ireland) on an Aglient Bioanalyser 2100 and using a Nanodrop spectrophotometer (Nanodrop Technologies, Wilmington, DE, USA), respectively. All RNA samples displayed RNA integrity numbers (RIN) greater than 8 and thus were deemed to be of suitable quality for subsequent RNA-sequencing. Individual cDNA libraries were prepared from each separate liver RNA sample for cattle divergent for RFI across each breed and dietary phase, using the Illumina TruSeq stranded mRNA sample prep kit (Illumina, San Diego, CA, USA) according to the manufacturer’s instructions. Resultant cDNA libraries were validated using the DNA 1000 Nano Lab Chip kit on the Aglient Bioanalyser 2100. Sequencing was subsequently undertaken on an Illumina HiSeq 2500 sequencer. All sequencing data used in this study are publicly available in NCBI’s Gene Expression Omnibus and can be accessed through GEO ID GSE111464.

Quality control of sequencing reads was undertaken using FastQC (v 0.11.5; [62]), followed by removal of sequencing adapters and any low quality reads using Cutadapt (v 1.13; [63]). Trimmed sequencing reads were mapped to the bovine reference genome (ARS-UCD1.2; [64]) and also quantified using STAR (v.2.5.1; [65]). Differential expression was undertaken using the edgeR package within the R environment [66]. Within edgeR, gene expression reads were estimated as Counts Per Million (CPM) and genes which presented with at least 1 CPM in at least half of the samples were retained for differential expression analysis. Differentially expressed genes were identified for each of the main contrasts of RFI phenotype (Low-RFI versus High-RFI), breed (Charolais versus Holstein-Friesian) and dietary source (high concentrate versus zero-grazed grass). The model for differential expression due to a given contrast (e.g., RFI) contained the other two contrasts as main effects (e.g., breed and diet).

### Co-expression network analysis

**Fig. 7:**
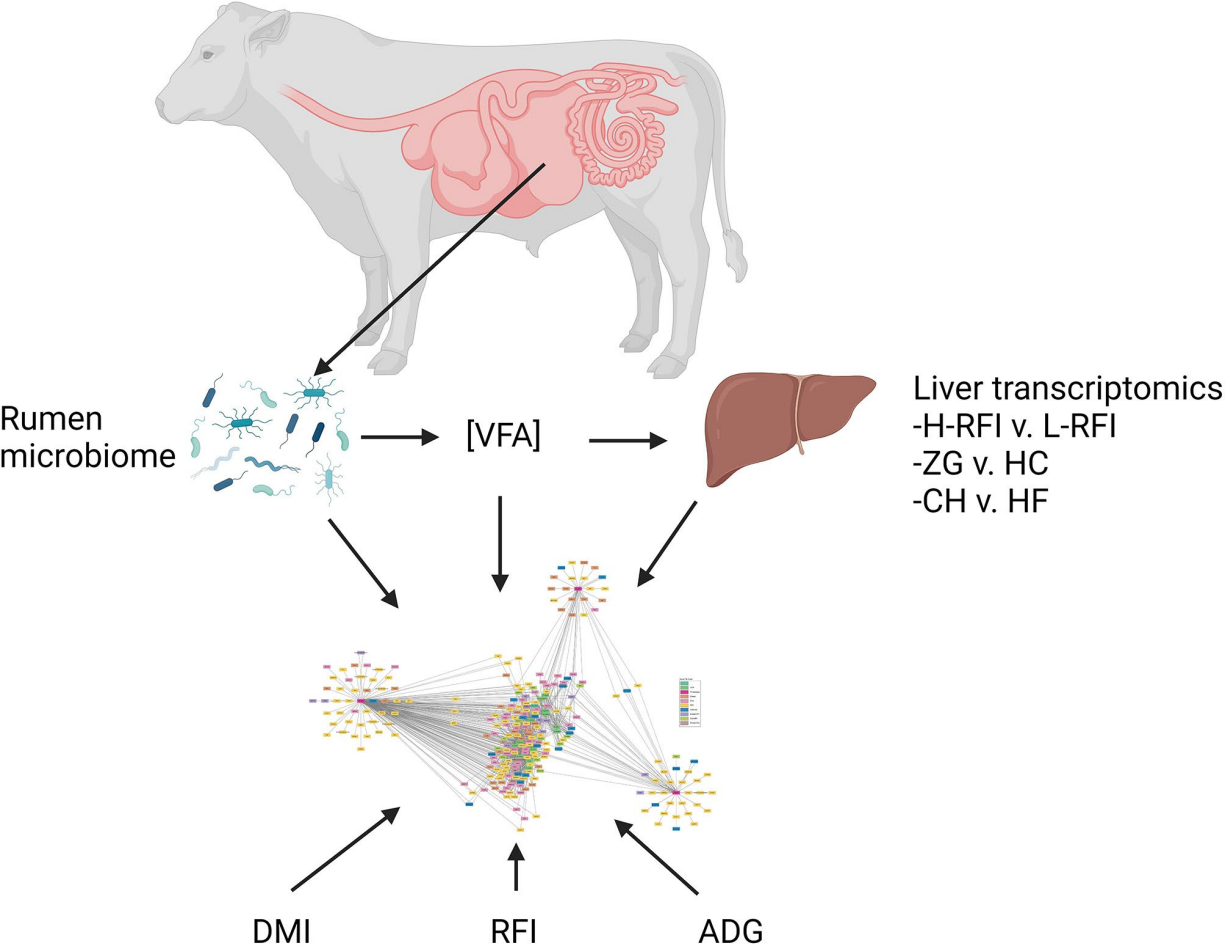
Overview of data used for co-expression network analysis. ([VFA]: volatile fatty acid concentrations; H-RFI: High-RFI; L-RFI: Low-RFI; ZG: zero-grazed grass diet; HC: high concentrate diet; CH: Charolais; HF: Holstein-Friesian; DMI: dry matter intake; RFI: residual feed intake; ADG: average daily gain)

The following datasets were utilised for the gene co-expression network analysis: liver transcriptomics; 16 S rumen microbial abundance taxonomy; VFA concentrations; and phenotype data (DMI, RFI and ADG; Fig. 7). From the differential expression analysis conducted in edgeR, the top 5% differentially expressed genes, for each of the three contrasts (RFI, breed, and diet) based on corrected *p*-value were selected for subsequent inclusion in the co-expression network analysis. Thus, nodes selected for subsequent co-expression analysis included: (i) genes differentially expressed based on the RFI contrast; (ii) genes differentially expressed based on the breed contrast; (iii) genes differentially expressed based on the diet contrast; (iv) ruminal microbial abundance; (v) VFA concentrations and; (vi) three phenotypes of interest: RFI, DMI and ADG. Significant connections (edges) between nodes were identified using the Partial Correlation and Information Theory (PCIT) algorithm [67]. The PCIT algorithm determines the significance of the correlation between a pair of nodes after accounting for all other nodes within the network [67]. The resultant network of co-expressed genes was imported into Cytoscape software [68] for visualisation. In order to assign biological annotation of the generated network, co-expression network analysis results were further evaluated for functional enrichment using Gprofiler and David gene ontology.

### Stepwise regression and correlation analyses

To determine the independent rumen microbial predictors of RFI, DMI and ADG, a stepwise regression analysis was conducted. This analysis was undertaken in SAS (version 9.4) using the REG procedure, incorporating the three phenotypes examined in this study as well as any microbe directly connected to the phenotypes, based on the results from the co-expression network analysis within the model and utilising R^2^ as the selection criteria. A correlation analysis was undertaken on the aforementioned phenotype and microbe variables using the CORR procedure of SAS. Additionally, a hierarchical clustering analysis was performed between the phenotypes and first neighbour microbial connections using PermutMatrix (version 1.9.4; http://www.atgc-montpellier.fr/permutmatrix/, [69]).

## Electronic supplementary material

Below is the link to the electronic supplementary material.

**Additional Table 1**: Microbial taxa identified within the rumen fluid and included within the co-expression network analysis. **Additional Table 2**: Top 5% differentially expressed genes for each RFI, breed and diet contrasts in liver tissue. **Additional Table 3**: First neighbours for each of the three phenotypes examined (DMI, RFI and ADG). **Additional Table 4**: First neighbours for microbial taxa directly connected to each of the three phenotypes examined (DMI, RFI and ADG)

## Data Availability

The transcriptomic datasets utilised for this study can be found in the NCBI’s Gene Expression Omnibus (GEO) database [https://www.ncbi.nlm.nih.gov/geo/] (GEO accession ID: GSE111464). Sequence files related to the 16s sequencing are available in NCBI Sequence Read Archive (Accession no. PRJNA483745).

## Abbreviations

ADG: Average daily gain
CPM: Counts per millions
DMI: Dry matter intake
DPBS: Dulbecco’s phosphate buffered saline
GEO: Gene expression omnibus
H1: High concentrate diet during the growing phase
H2: High concentrate diet during the finishing phase
HC: High concentrate
RFI: Residual feed intake
RIN: RNA integrity number
VFA: Volatile fatty acid
ZG: Zero-grazed grass diet during the growing phase
